# *In-Situ* Effects of Simulated Overfishing and Eutrophication on Benthic Coral Reef Algae Growth, Succession, and Composition in the Central Red Sea

**DOI:** 10.1371/journal.pone.0066992

**Published:** 2013-06-19

**Authors:** Christian Jessen, Cornelia Roder, Javier Felipe Villa Lizcano, Christian R. Voolstra, Christian Wild

**Affiliations:** 1 Coral Reef Ecology Group (CORE), Leibniz Center for Tropical Marine Ecology (ZMT), Bremen, Germany; 2 Red Sea Research Center, King Abdullah University of Science and Technology (KAUST), Thuwal, Saudi Arabia; 3 Faculty of Biology and Chemistry, University of Bremen, Bremen, Germany; The Australian National University, Australia

## Abstract

Overfishing and land-derived eutrophication are major local threats to coral reefs and may affect benthic communities, moving them from coral dominated reefs to algal dominated ones. The Central Red Sea is a highly under-investigated area, where healthy coral reefs are contending against intense coastal development. This *in-situ* study investigated both the independent and combined effects of manipulated inorganic nutrient enrichment (simulation of eutrophication) and herbivore exclosure (simulation of overfishing) on benthic algae development. Light-exposed and shaded terracotta tiles were positioned at an offshore patch reef close to Thuwal, Saudi Arabia and sampled over a period of 4 months. Findings revealed that nutrient enrichment alone affected neither algal dry mass nor algae-derived C or N production. In contrast, herbivore exclusion significantly increased algal dry mass up to 300-fold, and in conjunction with nutrient enrichment, this total increased to 500-fold. Though the increase in dry mass led to a 7 and 8-fold increase in organic C and N content, respectively, the algal C/N ratio (18±1) was significantly lowered in the combined treatment relative to controls (26±2). Furthermore, exclusion of herbivores significantly increased the relative abundance of filamentous algae on the light-exposed tiles and reduced crustose coralline algae and non-coralline red crusts on the shaded tiles. The combination of the herbivore exclusion and nutrient enrichment treatments pronounced these effects. The results of our study suggest that herbivore reduction, particularly when coupled with nutrient enrichment, favors non-calcifying, filamentous algae growth with high biomass production, which thoroughly outcompetes the encrusting (calcifying) algae that dominates in undisturbed conditions. These results suggest that the healthy reefs of the Central Red Sea may experience rapid shifts in benthic community composition with ensuing effects for biogeochemical cycles if anthropogenic impacts, particularly overfishing, are not controlled.

## Introduction

Both global stressors, such as emerging climate change resulting in ocean warming and acidification, and local factors are critically threatening coral reefs. Two of the most significant local stressors are eutrophication and overfishing [1].

Eutrophication stems from the over-enrichment of nutrients in water bodies. Sources of eutrophication in coastal marine environments are often anthropogenic in nature and include agriculture runoff, human sewage, urban waste, industrial effluent, and fossil fuel combustion [2]. Scleractinian corals, the primary reef ecosystem engineers [3], are mostly negatively impacted by eutrophication. The effects of eutrophication vary from reducing growth [4], [5] and calcification rates, [6], [7] to impairing reproduction [4], [8], lowering bleaching resistance [9], and advancing coral disease [10]. Algae is also affected by increased nutrient levels. Among those affected can be crustose coralline algae (CCA) [11]–[13], an important settlement substrates for corals [14], as well as turf and macroalgae [15]–[18].

Overfishing is the second local stressor simulated in this study. It has caused more than 90% worldwide decline of predators [19], and this lack of predators in an ecosystem has dramatic cascading effects. For example, in kelp forests, sea urchin populations exploded and led to immense deforestation following the removal of apex predators by fishing [19], [20]. In coral reefs, protection from overfishing can mitigate starfish outbreaks [21] and healthy herbivorous fish communities support higher resilience since they limit growth and establishment of algal communities [22]. Herbivore grazing in coral reefs helps maintain low algal turf growths, reduces the number and duration of coral-algal interactions, and increases space for coral settling by promoting encrusting coralline algae growth over macroalgae [23].

The pressures of eutrophication, overfishing and a combination thereof can cause benthic algae proliferation [24]. Once macroalgae are well established in a reef, herbivorous fish recruitment can be impeded by their natural avoidance of reef patches with high densities of macroalgae [25]. Macroalgae also compete for space with encrusting coralline algae, resulting in diminished coral larvae recruitment [26], [27], and the frequency and intensity of interactions between corals and algae can also increase [28]. As a consequence, excessive algal growth can lead to a reduction in coral recruitment [29] and can directly impact corals via allelochemicals [30]–[33] or decrease O_2_ availability in the direct vicinity [34]–[36]. In addition to a reduction in habitat complexity [37], the change in benthic community composition towards algal dominance also leads to an increase in algae-derived dissolved organic carbon (DOC) [38], [39]. Higher concentrations of DOC are known to stimulate microbial growth and metabolism [38]–[40] which in turn can negatively affect corals, presumably by unbalancing the coral-associated microbial community whose growth concomitantly generates hypoxic reef conditions [34]–[36], [41], [42].

Benthic algae can be useful bioindicators due to their fast growth and turnover rates [43], [44]. The predictions of the Relative Dominance Model (RDM) by Littler and Littler [45], state that a high cover of CCA over turf and frondose macroalgae is generally found in reef environments with elevated nutrient levels and an intact herbivorous community. Higher relative abundances of turf algae may indicate low nutrient and low grazing levels, while abundant frondose macroalgae represent the worst scenario, a combination of high nutrient and low herbivory levels. Until today, only limited support exists for this model. Though numerous studies compared the individual and combined effects of herbivory and nutrient availability on benthic algal community composition [11]–[13], [24], [46]–[51], many of these studies were of limited duration. While the RDM is still under debate [13], [17], [24], [46], [49], no comparative studies exist for the Red Sea, and the individual effects of nutrient enrichment and herbivory exclusion have received little attention in this area (bottom-up: [52], [53]; top-down: [54]–[56]). Meanwhile, emerging coastal development together with overfishing and land-derived nutrient run-off are threatening many healthy Red Sea coral reefs, particularly around the fast developing and wealthy Jeddah region [1], [57].

The study presented was designed to answer the following questions: (1) What influence, if any, do increased nutrient availability (bottom-up factor) and herbivore exclusion (top-down factor) have on benthic algae development, in terms of dry mass, organic carbon (C) and nitrogen (N) production, O2 consumption, and community composition? (2) Which factor, bottom-up or top-down, demonstrates a larger effect in this context? (3) Does the availability of light compound the benthic algae development? To answer these questions, we conducted an in-situ experiment in an offshore reef in the Central Red Sea over 4 months, simulating the individual and combined effects of eutrophication and overfishing.

## Materials and Methods

### Ethics Statement

The study site of Al Fahal reef does not fall under any legislative protection or special designation as a marine/environmental protected area. No special permit is required for the inshore coastal, reef, and intertidal areas around Thuwal. The Saudi Coast Guard Authority under the auspices of KAUST University issued sailing permits to the site, which included sample (algae) collection.

### Study Site

The study was carried out from June to September 2011 over a period of 16 wks at the patch reef Al Fahal about 13 km off the Saudi Arabian coast in the Central Red Sea (N22.18.333, E38.57.768; Figure S1). Al Fahal is located >80 km to urban areas (next large city is Jeddah, >3 Mio inhabitants), with only a small village (Thuwal) located on shore. Neither are river deltas located in this region nor is any land of the surrounding region allocated for agriculture. This reef was chosen in particular, due to its relatively large distance from shore and minimal impacts from land-based nutrient import and large-scale fishing.

### Benthic Cover

Benthic reef community composition was assessed using the linear point intercept (LPI) method [58]. Benthic coverage was classified every 0.5 m along a 70 m transect that ran along the investigated reef site into the following categories: hard coral, soft coral, coral rubble (<20 cm), rock (bare substrate and rubble >20 cm), CCA, macroalgae (erected non-filamentous algae, e.g. Padina, Halimeda, Turbinaria, Ulva), filamentous algae (>2 mm), and other.

### Cage Setups

Sixteen polyvinyl chloride (PVC) frames (50×75 cm) were deployed in the reef at 5–6 m water depths along a 70 m transect with 2–5 m distance in between. Each frame was equipped with 12 terracotta tiles, each with 100 cm^2^ surface area. Prior to the start of the experiment, the tiles were autoclaved to remove any interfering compounds that could have accumulated during tile production and transported to the study site in a sealed plastic bag to avoid contamination. Tiles were installed pairwise on top of each other with the unglazed sides facing outside, resulting in an upper (light-exposed) and lower (shaded) tile. To avoid excessive sedimentation, tiles were installed at an angle of 45 degrees approximately 10 cm above the reef substrate using stainless steel screws, nuts, and washers. Four different treatments were applied to the frames (each with a replication of n = 4): (1) control (only the equipped frame), (2) fertilizer tubes (see nutrient enrichment section), (3) cage (hemispherical zinc galvanized cages with a mesh size of 4 cm and a diameter of 100 cm), and (4) a combination of cage and fertilizer tubes. The cages served to exclude larger herbivores; smaller fish (e.g. small parrotfish, wrasses, and surgeonfish) were still able to gain access to the tiles. High numbers of mobile grazing invertebrates (e.g. crustaceans, polychaetes, or gastropods) were not observed in any of the cages. Cage controls were not used, since studies showed that similar cages even with a lower mesh size did not affect water movement, light availability, and sedimentation rates [48], [59], [60].

Nutrient enrichment was simulated by deploying 4 fertilizer tubes around the frame, consisting of perforated PVC tubes filled with Osmocote fertilizer (Scotts; 15% total nitrogen as nitrate & ammonium, 9% phosphate as phosphoric pentoxide, and 12% potassium oxide) embedded in 3% agarose. Fertilizer dry mass was 580 g per frame. Fertilizer was deployed once without replenishments, but regular monitoring of inorganic nutrient concentrations assured continuous enrichment levels (actual values will be presented in the results section).

On each of the 5 sampling events one pair of tiles (light-exposed and shaded) was collected per frame, after 1, 2, 4, 8, and 16 wk(s) using SCUBA. All tiles were pre-scored and first divided in half (each 50 cm^2^; an area which had been chosen from the asymptote of species-area curves by Hixon and Brostoff [61]) and then wrapped separately in ziplock bags. They were brought on board within 30 min where half of them were immediately flash frozen in liquid nitrogen for subsequent microbial analyses (results reported elsewhere), while the other half was handled as described below.

### Incubations

O_2_ consumption rates were measured after a modified method by Wild et al. [36]. Tiles were stored without air bubble inclusions in 1 L airtight incubation glass jars, that were kept in 4 large (70 L volume), opaque polyethylene (PE) containers filled with reef water to keep samples at constant ambient temperatures during incubations (monitored with Onset HOBO pendant temperature loggers in each container). Incubations were run in closed and dark containers. Temperature differences between *in-situ* temperatures (measured at PVC frames) and incubation jars ranged from 0.5 to 1.6°C). Net O_2_ consumption rates were calculated for each incubation jar by dividing the difference between initial and end O_2_ concentrations by the incubation duration (1.5–1.7 h) and corrected by subtracting mean O_2_ consumption rate of 4 seawater controls without tiles. During incubations, the boxes were carefully moved by hand every 5 minutes on one side to mix the water inside the jars. O_2_ measurements were carried out using a Hach O_2_ probe (Hach HQ40d) that was placed a few cm above each tile in the incubation jars. All samples were stored on ice until further processing.

### Response Variables on the Tiles

Light-exposed and shaded tiles were rinsed with fresh water to remove salt, attached sediment, and mobile invertebrates, resulting in light-exposed tiles that were almost exclusively covered with algal material with very rare invertebrate cover. Tiles were then photographed with a digital camera, before algal cover was carefully removed by using spatula and scalpel (only light-exposed tiles). The removed algae cover was dried in an oven at 37°C to constant weight, and dry mass (non-decalcified) was measured with a precision balance (Mettler Toledo XS205, accuracy: 0.01 mg). Until further processing, samples were kept dry at 37°C.

To quantify the proportional coverage of functional groups on the light-exposed and shaded tiles, 100 points were randomly overlaid on the digital picture of each tile using the software Coral Point Count with Excel extensions (CPCe) 4.1 [62]. Applied categories were: open space (non biotic cover or bare terracotta surface), filamentous algae (≥2 mm), crustose coralline algae (CCA), green crusts (non-coralline light green crusts), red crusts (non-coralline red crusts, e.g. *Peyssonnelia* spp.), brownish crusts (non-coralline dark-green and brownish crusts, e.g. filamentous algae <2 mm), cyanobacteria (whitish & mucilaginous), red macroalgae (fleshy upright red algae), and invertebrates (sessile forms).

For the elemental analyses of algae tissue, samples were homogenized using mortar and pestle and subsequently either acidified (organic C) or directly measured (N) with a EuroVector elemental analyzer (EURO EA 3000). Carbon and nitrogen contents were derived from calculation using elemental standards (apple leaf standard; Hekatech: HE34010100; analytical precision ≤0.1% (N) and ≤0.6% (C) of the standard value). Isotopic analysis of δ15N signatures of dried algal material relative to atmospheric nitrogen was run with an isotope ratio mass spectrometer (Finnigan Corp., San Jose, CA).

One of the 4 cage barriers deployed in the combined treatment seemed to have been breached by large herbivores, as evidenced by tile appearance and cage warping; the data (i.e. algal dry mass, organic C, N, O_2_ consumption, and functional group assemblages) from said replicate were removed from the subsequent analysisafter application of Grubb’s outlier tests.

### Water Parameters

Directly before sampling of the tiles, samples of ∼5 L seawater (in total n = 80; 40 enriched and 40 non-enriched) were collected with large ziplock bags directly from above each frame. From this stock, 1000 mL were filtered on untreated Whatman-GF/F filters (Chlorophyll *a* (Chl *a*)) and 1000–2500 mL on pre-combusted and pre-weighted filters for particulate organic matter (POM). Due to laboratory mishap there were no samples for wk 1 for particulate organic nitrogen (PON) and only 1 sample from 1 treatment for particulate organic carbon (POC). Elemental analyses of N and organic C of POM were performed using an EuroVector elemental analyzer (EURO EA 3000). The remaining filtrate was further used for nutrient (50 mL) and dissolved organic matter (DOM) measurements (40 mL). Analyses of dissolved inorganic nitrogen (DIN = NH_4_^+^+NO_3_^−^+NO^2−^) and soluble reactive phosphorous (SRP = PO_4_^3−^) were performed using a continuous flow analyzer (FlowSys Alliance Instruments). Dissolved organic matter (DOM) measurements were carried out with the Teledyne Tekmar Apollo 9000 Combustion TOC/TN Analyzer. Chl *a* filters were stored at −20°C prior to acetone-extraction (90%) and measured fluorometrically according to the method described in Environmental Protection Agency (EPA) 445.0 [63].

Over the study period, temperature data were continuously measured (at 5 minutes intervals) at all PVC frames using HOBO pendant and Pro v2 loggers (Onset Computer Corporation, Pocasset, MA).

### Herbivore Biomass

Visual surveys of herbivorous fish and sea urchins were carried out along the 70 m long transect of the frames in 5 m water depth with 4 replicates from June to July 2011. The fish surveys were conducted at noon between 11∶15 am and 12∶15 pm, 2.5 m left and 2.5 m right from the 70 m transect line, surveying a total area of 350 m^2^. All herbivorous species ≥5 cm were counted, their size estimated, and grouped in one of 4 size classes (5–10 cm, 10–20 cm, 20–30 cm, and 30–40 cm). Species identification followed Randall [64], Debelius [65], and Lieske and Myers [66]. Classifying fish into herbivorous and non-herbivorous groups was based on Randall [64], Khalaf and Disi [67], Lieske and Myers [66], and own observations of grazing species (Table S1). Classification of herbivores took place according to their ability to remove algal material from the reef and not on their physiological ability to digest algal material [68]. Biomass of herbivorous fish was calculated on basis of the average length of the size class following length-weight ratios of the species or when not available of their family published by Green and Bellwood [69] and in FishBase [70].

No sea urchin species were observed during the 4 daytime surveys, so the sea urchin survey was conducted after the sun had fully set at 8 pm. The survey area was reduced to 1 m in width, resulting in a total surveyed area of 70 m^2^. All sea urchins encountered along a 1 m polyethylene (PE) bar were counted and their test diameters were measured with a caliper to the nearest cm. Biomass was calculated on the basis of published length-weight relationships [71]–[73].

### Statistical Data Analysis

Data from nutrient concentrations were analyzed using 2-sided t-tests. Water parameter data of Chl *a*, PON, POC, DON, DOC, as well as algal dry mass, organic C, N content, C_org_/N ratio, δ15N signatures of exposed tile cover, and O_2_ consumption rates (log transformation of values from light-exposed tiles) were analyzed using a 3-factorial ANOVA with backward stepwise deletion of variables, containing cage (present/absent), fertilizer (present/absent), time (5 sampling times), and their interactions as fixed factors. Functional algal group compositions were analyzed using a 3-factorial generalized linear model (GLM) with quasibinomial distribution and logit function. ANOVA and GLM analyses were carried out with the R statistical software version 2.15.2 [74]. To meet test assumptions of normal distribution and homoscedasticity, data of algal dry mass were log(x+1) transformed.

## Results

### Reef Background Parameters

Linear point intercept surveys revealed coral as dominating benthic feature (49%; with 32% hard coral and 17% soft coral), followed by rock (27%), coral rubble (13%), CCA (7%), filamentous algae (2%), and other (2%). Macroalgae were not observed.

During 4 transect surveys, 532 herbivorous fish were counted. Sixteen different species from 8 families with a total abundance of 0.4±0.1 ind. m^−2^ (mean±SE) and biomass of 22.4±8.0 g m^−2^ were found. Scaridae (8.9 g m^−2^) and Acanthuridae (9.8 g m^−2^) had the largest biomass (Table S1). During the sea urchin survey, 120 individuals of 4 species (*Echinometra mathaei*, *Echinothrix calamaris*, *Eucidaris metularia*, and *Heterocentrotus mammillatus*) were counted. Sea urchins exhibited a mean total abundance of 1.71 ind. m^−2^ and a biomass of 37.5 g m^−2^ (Table S2).

### Experimental Background Parameters

**Figure 1:**
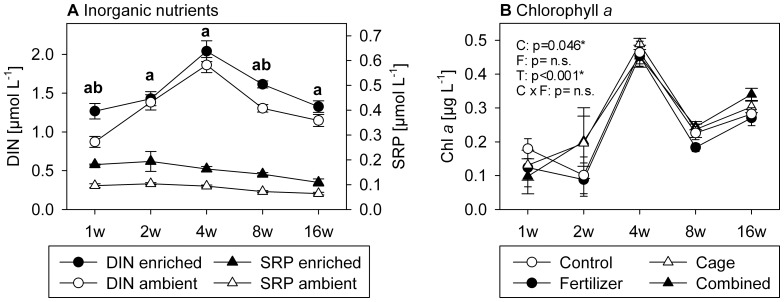
Inorganic nutrient (A) and Chlorophyll *a* (B) concentrations. A: Inorganic nutrient concentrations (µmol L^−1^; means±SE) in the nutrient enrichment treatments (fertilizer & combined) and the non-enriched treatments (control & cage). Small letters (a for SRP; b for DIN) indicate statistical significant differences between enriched and non-enriched plots of p<0.05 (t-test). DIN: dissolved inorganic nitrogen; SRP: soluble reactive phosphate. B: Chlorophyll *a* concentrations (µg L^−1^, means±SE) from water samples taken directly above the tile setups at all 5 sampling times. P-values were calculated from 3-factorial ANOVA and originate from analysis across the whole study period (see Table 1 for full results). P-values were tagged as n.s. ( = not significant), when the model reduction step excluded the corresponding factor(s).

The fertilizer and combined treatment led to an increase in DIN concentrations in the water column above the frames with significant differences for wk 1 and 4 in comparison to the non-enriched treatments. DIN concentrations changed over time with a peak after 4 wks (Figure 1A). In contrast, SRP concentrations remained rather constant, but enriched and non-enriched treatments significantly differed over all sampling times (Figure 1A).

**Figure 2:**
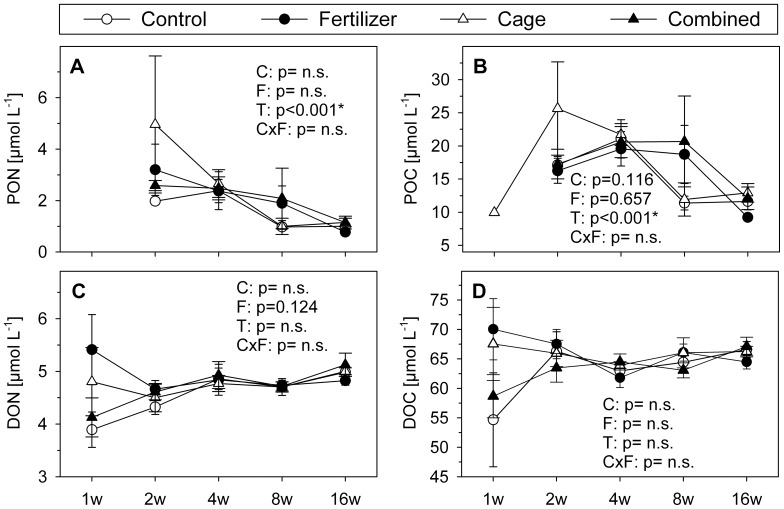
Concentrations of particulate and dissolved organic matter. Particulate (mg cm^−2^, means±SE) and dissolved organic matter concentrations (µmol L^−1^, means±SE) in water samples taken directly above the installations A: particulate organic nitrogen (PON), B: particulate organic carbon (POC), C: dissolved organic nitrogen (DON), and D: dissolved organic carbon (DOC). Shown are data of all treatments for all 5 sampling times. P-values were calculated from 3-factorial ANOVA and originate from analysis across the whole study period (see Table 1 for full results). Abbreviations: C = Cage, F = Fertilizer, T = Time. Missing values of 1wk for PON and POC resulted from insufficient algal dry mass for analysis. Shown P-values originate from analysis across the whole study period. P-values were tagged as n.s. ( = not significant), when the model reduction step excluded the corresponding factor(s).

Only Chl *a* (Figure 1B), but not POM (Figure 2A and 2B, Table 1) and DOM (Figure 2C and 2D, Table 1) concentrations in the water column directly above the setup were influenced by the treatments. Chl *a* values above the caged treatments were significantly higher than those of the non caged treatments (Figure 1B). Chl *a* together with PON and POC concentrations were significantly influenced by time. Chl *a* levels peaked after 4 wks and increased again after 16 wks following a drop at wk 8, while PON and POC concentrations declined and DON and DOC concentrations remained constant.

**Table 1:** Results of the 3-factorial ANOVA of the water parameters.

	Chlorophyll *a*			PON			POC			DON			DOC		
	df	*F*	*P*	df	*F*	*P*	df	*F*	*P*	df	*F*	*P*	df	*F*	*P*
C	1	4.17	0.046*	–	–	–	1	2.56	0.116	–	–	–	–	–	–
F	–	–	–	–	–	–	1	0.20	0.657	1	2.42	0.124	–	–	–
T	4	43.97	0.000*	4	5.10	0.001*	4	6.56	0.000*	–	–	–	–	–	–
C×F	–	–	–	–	–	–	–	–	–	–	–	–	–	–	–
T×C	4	2.43	0.057	–	–	–	–	–	–	–	–	–	–	–	–
T×F	–	–	–	–	–	–	3	3.63	0.019*	–	–	–	–	–	–
T×C×F	–	–	–	–	–	–	–	–	–	–	–	–	–	–	–

Response variables (1^st^ row) are chlorophyll *a*, particulate organic nitrogen (PON), particulate organic carbon (POC), dissolved organic nitrogen (DON), dissolved organic carbon (DOC). Independent factors (1^st^ column) are Cage (C), Fertilizer (F), and Time (T). Significant results are indicated by asterisks. P-values of 0.000 symbolize values <0.001. Dashes represent factors that have been excluded by the model reduction.

### Effects on Tile Cover

#### Nutrient enrichment effects

**Figure 3:**
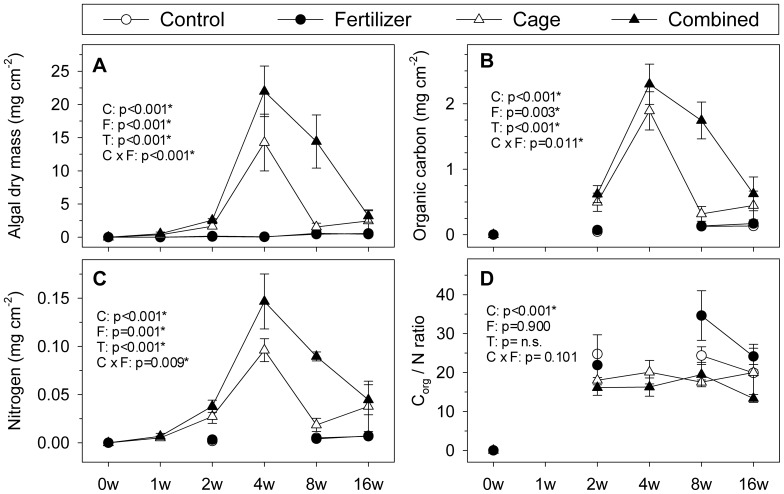
Development of algal dry mass (A), organic carbon (B), nitrogen content (C), and organic C_/_N ratio (D) on light-exposed tiles. Shown are means±SE of all treatments in mg cm^−2^ over the 5 sampling points after 1, 2, 4, 8 and 16 wk(s). P-values were calculated from a 3-factorial ANOVA and originate from analysis across the whole study period (see Table 1 for full results). P-values were tagged as n.s. ( = not significant), when the model reduction step excluded the corresponding factor(s). Missing connections between data points are due to insufficient algal material for analysis. Abbreviations: C = Cage, F = Fertilizer, T = Time.

**Figure 4:**
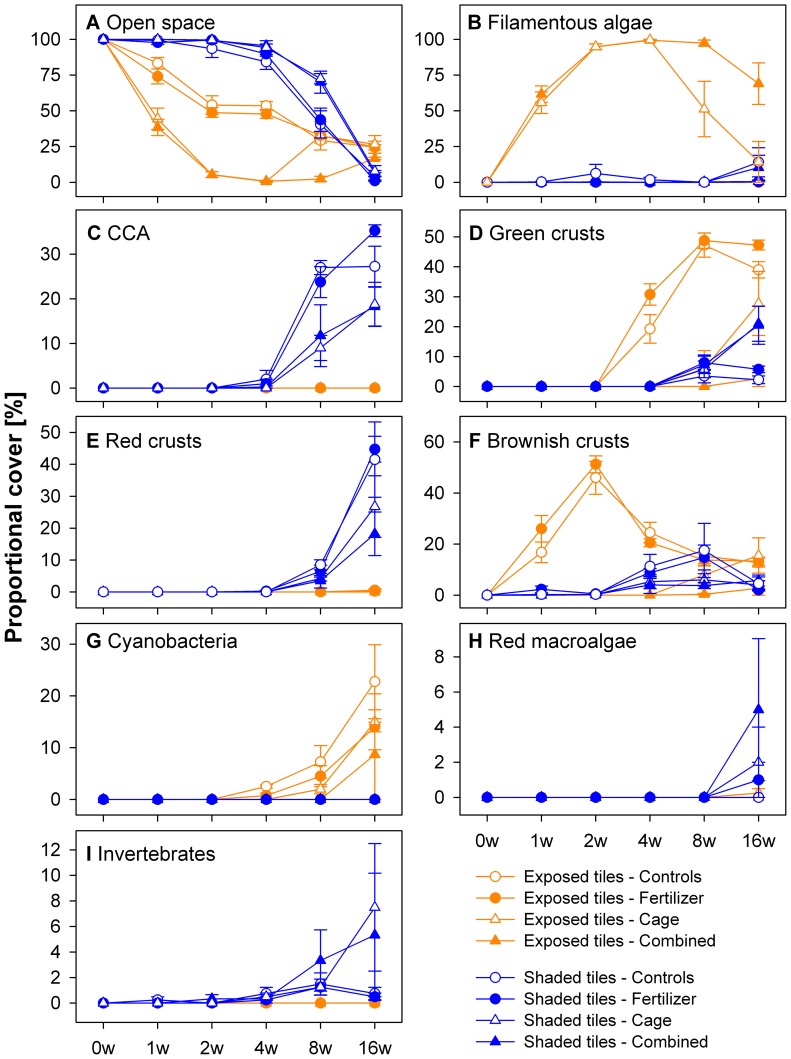
Percent cover of functional groups on light-exposed (orange) and shaded tiles (blue). Shown is the proportional cover (means±SE) over the study period of 4 months of functional groups in the 4 treatments: control, fertilizer, cage, and combined. See Tables 3 and 4 for statistical results.

**Figure 5:**
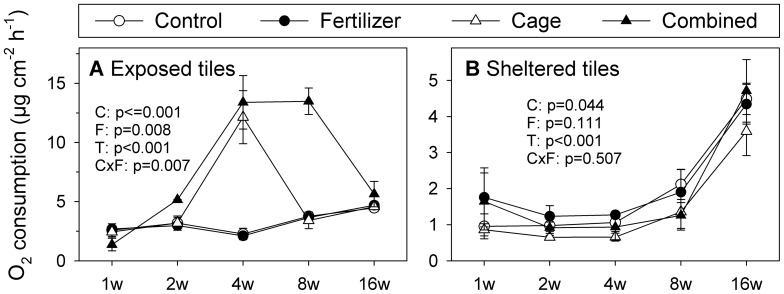
O_2_ consumption rates. O_2_ consumption rates of A: light-exposed tiles and B: shaded tiles in µg cm^−2^ h^−1^ (means±SE). P-values are calculated from 3-factorial ANOVA and originate from analysis across the whole study period (see Table 5 for full test results). Significant p-values (p<0.05) are indicated by asterisks. Abbreviations: C = Cage, F = Fertilizer, T = Time.

Nutrient enrichment had no effect on algal dry mass, organic C, and N on the light-exposed tiles compared to controls when applied individually (Figure 3, Table 2). This result is contrasted with the δ15N values, which were significantly decreased in the enriched treatments compared to controls (Figure S3; Table S3). Additionally, benthic cover was not significantly altered by nutrient enrichment except for decreasing cyanobacteria cover on the light-exposed tiles (Figure 4G, Table 3) and green crusts on the shaded tiles (Figure 4D, Table 4) compared to controls. Furthermore, O_2_ respiration rates of the light-exposed and shaded tiles did not significantly differ between controls and nutrient addition (Figure 5).

**Table 2:** Results of the 3-factorial ANOVA of the algal parameters.

	Algal dry mass			Algal C organic			Algal N			C_org_/N		
	df	*F*	*P*	df	*F*	*P*	df	*F*	*P*	df	*F*	*P*
C	1	253.83	0.000*	1	30.49	0.000*	1	37.13	0.000*	1	13.38	0.000*
F	1	15.42	0.000*	1	11.87	0.000*	1	12.10	0.001*	1	0.02	0.900
T	4	26.63	0.000*	3	32.41	0.000*	4	33.24	0.000*	–	–	–
C×F	1	15.44	0.000*	1	9.50	0.000*	1	7.51	0.009*	1	2.83	0.101
T×C	4	28.71	0.000*	2	2.36	0.000*	2	0.97	0.387	–	–	–
T×F	4	2.75	0.037*	3	2.94	0.037*	4	2.81	0.039*	–	–	–
T×C×F	4	4.46	0.003*	2	4.08	0.003*	2	3.10	0.057	–	–	–

Response variables (1^st^ row) are algal dry mass, algal organic C, algal N of the exposed tiles. Independent factors (1^st^ column) are Cage (C), Fertilizer (F), and Time (T). Algal dry mass data were log (x+1) transformed to meet parametric assumptions. Significant results are indicated by asterisks. P-values of 0.000 symbolize values <0.001. Dashes represent factors that have been excluded by the model reduction.

**Table 3:** Results of 3-factorial generalized linear model (GLM; binomial distribution and logit function) of functional algal groups of light-exposed tiles.

	Open space			Filamentous algae			CCA		
	df	*F*	*P*	df	*F*	*P*	df	*F*	*P*
C	1	210.20	0.000*	1	693.74	0.000*	x	x	x
F	1	9.77	0.002*	1	30.99	0.000*	x	x	x
T	4	44.04	0.000*	4	31.55	0.000*	x	x	x
C×F	1	6.07	0.017*	–	–	–	x	x	x
T×C	4	27.34	0.000*	–	–	–	x	x	x
T×F	4	0.50	0.739	4	4.45	0.003*	x	x	x
T×C×F	4	4.83	0.002*	–	–	–	x	x	x
	**Green crusts**			**Red crusts**			**Brownish crusts**		
	**df**	***F***	***P***	**df**	***F***	***P***	**df**	***F***	***P***
C	1	192.46	0.000*	1	23.29	0.000*	1	250.90	0.000*
F	1	0.19	0.667	1	2.04	0.159	1	0.44	0.512
T	4	96.84	0.000*	4	14.44	0.000*	4	16.82	0.000*
C×F	1	30.13	0.000*	–	–	–	1	14.21	0.000*
T×C	4	7.97	0.000*	–	–	–	4	23.10	0.000*
T×F	4	0.49	0.746	–	–	–	4	1.04	0.394
T×C×F	4	0.12	0.973	–	–	–	4	0.11	0.978
	**Cyanobacteria**			**Red algae**			**Invertebrates**		
	**df**	***F***	***P***	**df**	***F***	***P***	**df**	***F***	***P***
C	1	13.08	0.000*	1	24.91	0.000*	x	x	x
F	1	8.93	0.004*	1	20.12	0.000*	x	x	x
T	4	51.75	0.000*	4	14.00	0.000*	x	x	x
C×F	1	0.17	0.677	–	–	–	x	x	x
T×C	4	1.73	0.157	–	–	–	x	x	x
T×F	4	0.11	0.980	–	–	–	x	x	x
T×C×F	4	0.43	0.789	–	–	–	x	x	x

Response variables are shown in the 1^st^ row and in the first column the independent factors: Cage (C), Fertilizer (F), and Time (T). Significant results are indicated by asterisks. P-values of 0.000 symbolize values <0.001. Dashes represent factors that have been excluded by the model reduction and ‘x’ stands for insufficient data for analysis.

**Table 4:** Results of 3-factorial generalized linear model (GLM; binomial distribution and logit function) of functional algal groups of light shaded tiles.

	Open space			Filamentous algae			CCA		
	df	*F*	*P*	df	*F*	*P*	df	*F*	*P*
C	1	39.64	0.000*	1	2.19	0.144	1	60.94	0.000*
F	1	0.88	0.354	1	2.90	0.094	1	0.01	0.909
T	4	225.73	0.000*	4	11.39	0.000*	4	150.47	0.000*
C×F	1	0.01	0.919	1	26.21	0.000*	1	2.60	0.113
T×C	4	0.39	0.816	4	0.35	0.844	4	2.90	0.030*
T×F	4	1.05	0.391	4	0.45	0.768	4	1.23	0.309
T×C×F	4	0.67	0.618	4	0.16	0.999	–	–	–
	**Green crusts**			**Red crusts**			**Brownish crusts**		
	**df**	***F***	***P***	**df**	***F***	***P***	**df**	***F***	***P***
C	1	40.47	0.000*	1	38.78	0.000*	1	9.53	0.003*
F	1	4.38	0.041*	1	2.84	0.098	1	0.43	0.512
T	4	66.75	0.000*	4	132.64	0.000*	4	15.76	0.000*
C×F	1	2.13	0.150	1	8.20	0.006*	1	0.07	0.797
T×C	4	3.18	0.020*	4	0.02	0.999	4	2.03	0.103
T×F	4	0.17	0.952	4	0.28	0.889	4	0.55	0.702
T×C×F	4	0.13	0.971	4	0.01	0.999	4	0.43	0.787
	**Cyanobacteria**			**Red algae**			**Invertebrates**		
	**df**	***F***	***P***	**df**	***F***	***P***	**df**	***F***	***P***
C	x	x	x	1	18.62	0.000*	1	14.16	0.000*
F	x	x	x	1	7.65	0.007*	1	0.13	0.721
T	x	x	x	4	21.06	0.000*	1	12.94	0.000*
C×F	x	x	x	1	2.32	0.133	2	0.71	0.403
T×C	x	x	x	–	–	–	2	2.22	0.078
T×F	x	x	x	–	–	–	2	0.87	0.488
T×C×F	x	x	x	–	–	–	2	0.09	0.984

Response variables are shown in the 1^st^ row and in the first column the independent factors: Cage (C), Fertilizer (F), and Time (T). Significant results are indicated by asterisks. P-values of 0.000 symbolize values <0.001. Dashes represent factors that have been excluded by the model reduction and ‘x’ stands for insufficient data for analysis.

#### Herbivore exclusion effects

In contrast, herbivore exclusion significantly increased algal dry mass, organic C, and N content and decreased the organic C/N ratio on the light-exposed tiles at all sampling times compared to the control treatment (Figure 3, Table 2). Furthermore, on the light-exposed tiles, filamentous algae grew exclusively in the caged treatments, while the cover of green (40% decrease compared to controls) and brownish crusts (50% decrease) and cyanobacteria (7% decrease) were significantly decreased (Figure 4B, 4D, 4F and 4G; Table 3). Shaded tiles revealed a very different picture; herbivore exclusion significantly enhanced cover of green crusts (20% increase compared to controls) (Figure 4D, Table 4) and invertebrates (7% increase), while red crusts (15% decrease) (Figure 4C, Table 4) and CCA (20% decrease) (Figure 4E, Table 4) were suppressed. Together with algal dry mass, O_2_ consumption rates increased when herbivores were excluded on the light-exposed tiles (Pearson correlation, r = 0.65, p<0.05), but no treatment effect was detectable for the shaded tiles (Table 5).

**Table 5:** Results of the 3-factorial ANOVA of O_2_ consumption of exposed and shaded tiles.

	Exposed Tiles			Shaded Tiles		
	df	*F*	*P*	df	*F*	*P*
C	1	35.15	0.000*	1	3.70	0.056
F	1	3.87	0.054	1	1.92	0.170
T	4	18.27	0.000*	4	39.84	0.000*
C×F	1	3.68	0.060	–	–	–
T×C	4	10.10	0.000*	–	–	–
T×F	4	1.68	0.006*	–	–	–
T×C×F	4	5.79	0.000*	–	–	–

Response variables are shown in the 1^st^ row. In the 1^st^ column are the independent factors: Cage (C), Fertilizer (F), and Time (T). Significant results are indicated by asterisks. P-values of 0.000 symbolize values <0.001 and dashes represent factors that have been excluded by the model reduction. n.s. = not significant.

#### Combined effects

The interaction of herbivore exclusion and nutrient enrichment was significant on the light-exposed tiles and further increased algal biomass in terms of algal dry mass, organic C, N, and O_2_ consumption rates compared to the cage treatment (Figures 3 and 5). Filamentous algae cover was increased by a further 50%, compared to cage treatments (Figure 4B, Table 3) and cyanobacteria decreased a further 5%. (Figure 4G, Table 3). Red crusts on the shaded tiles had their percent cover further reduced by 9% in the combined treatments compared to the cage treatments (Figure 4E, Table 4).

#### Temporal changes

The temporal patterns in the development of algal biomass in terms of dry mass, organic C, N, and O_2_ consumption rates on the light-exposed tiles were similar: while the non-caged treatments had no significant effects, the herbivore exclusion treatments exhibited a gradual increase of these data markers over the course of the first 4 wks of the study. Compared to the control, 300-fold, 7-fold, 8-fold, and 5-fold increases were observed in algal dry mass, organic C and N, and O_2_ consumption rates, respectively (Figures 3 and 5). This peak at wk 4 was followed by a drop to lower values in wks 8 and 16. The algal dry mass in the cage treatment decreased rapidly down to wk 2 levels, unlike the combined treatment, where the peak after wk 4 was even higher (500 times in algal dry mass, 9 times in organic C, 11 times in N, and 6 times in O_2_ consumption rates compared to the controls) and the decline was much less pronounced (Figures 3 and 5).

## Discussion

### Status of the Reef

High coral cover and lack of macroalgae at Al Fahal reef suggest a healthy reef [43] that ranks highly compared to Indo-Pacific reefs [75] and more closely to the pristine reefs from the northern Line Islands [76]. The rock and rubble proportion of the benthic cover of the reef may have originated from a recent bleaching event in the region [77]. Our measurements of herbivorous fish biomass (22 g m^−2^) were below the pristine reefs of Kingman (32 g m^−2^) [76], the average Indo-Pacific values (29 g m^−2^) [75], and data from recent studies in the Red Sea (63 g m^−2^ in 5 m water depth by Brokovich et al. [78] and 27 g m^−2^ by Khalil et al. [79]. However, other studies suggest that the measured biomass values of our study correspond to unfished reefs (e.g. [80], [81]). This is supported by the sea urchin biomass at our study site (38 g m^−2^), typical for unfished reefs [82], [83]. Ambient concentrations of SRP ranged under the thresholds of increased macroalgae growth of 1.0 µmol L^−1^ for DIN and 0.1 µmol L^−1^ for SRP proposed by Bell [84] & Lapointe [15], though these values are under discussion [17], [85] and many field studies have not found data supporting these thresholds [16], [47], [48], [51], [86]–[89]. In contrast, DIN ambient concentrations exceeded the threshold after the 1st wk. However, the low DOC and Chl *a* values (DOC: [41], [90], Chl *a*: [84]) suggest that the reef is little impacted by eutrophication.

### Effects of Treatments

Nutrient concentrations in the enriched treatments constantly exceeded ambient conditions and ranged above the suggested thresholds of Bell [84] and Lapointe [15], showing the successful enrichment. However, nutrient concentrations of the enriched treatments in this study are less enhanced than in similar experiments (e.g. [12], [13]). We assume that the large water sampling volumes and the concomitant dilution of samples prevented the detection of higher nutrient levels in the enrichment treatments. This view is supported by the Chl *a*, POM, and DOM concentrations in the water column just above the treatments that were not significantly influenced by fertilizer addition or other treatments.

#### Algal Biomass

Nutrient enrichment altered algal biomass on the light-exposed tiles only in interaction with herbivore exclusion in terms of algal dry mass, organic C, and N. However, it is likely that a larger effect of nutrient enrichment was masked by compensatory feeding by herbivores [12]. In contrast to the nutrient treatment, herbivore exclusion had an immediate and direct influence on most measured algal parameters, which was further extended by the combined treatment.

C and N removal rates are strongly connected to algal wet and dry mass. However, C and N data analyses provide a more neutral method than other biomass measures because values are independent of algal species and their calcified structures, if any, and permit greater comparability between studies, albeit data available are scarce. Only one recent study from the Egyptian Red Sea [55] showed N removal rates and their maxima were similar to the results found here. The consistently lower organic C/N ratio in the caged treatments indicates that herbivore preferentially graze on N rich algae [91]–[93], which did not accumulate outside the cages. Furthermore, C/N ratio data suggest that extra N provided by the fertilizer was directly used for growth and not stored in the algal tissue as previously reported for depleted but not for enriched algal tissue [94]. The uptake of extra N from the fertilizer could therefore not be proven by the C/N ratio, but by the isotope analysis. The δ15N ratio of the fertilizer was close to 0, and the incorporation of the fertilizer therefore should reduce the δ15N ratio of the algal material. This reduction could be shown in the enriched frames over the non-enriched frames (Figure S3; Table S3).

If not controlled, algal biomass can increase to huge quantities, in our experiment up to 19 mg cm^−2^ wk^−1^. This would be 190 t wk^−1^ if extrapolated to a reef of 1 km^2^.

Our findings from the Red Sea demonstrated that decreased herbivory has a stronger influence on algal biomass than increased nutrients, corresponding to the majority of comparative studies from reefs around the world that compared herbivory versus nutrient enrichment on algal growth (Australia: [86], [87], [95]; Caribbean: [12], [47], [48], [50]; Hawaii: [13], [60]; Guam: [11], [51]. Yet, other studies collected evidence that nutrient enrichment can also have larger and delayed influence on algal development and the ability of algae to overgrow corals [13], [96], [97].

Our data clearly show that nutrient enrichment alone was not able to increase algal biomass, even when the proposed threshold concentrations of 1.0 µmol L^−1^ of DIN and 0.1 µmol L^−1^ of SRP [15], [84] were exceeded for most of the study time. One may argue, that the ambient nutrient levels already saturated the nutrient needs of most algae and field and laboratory studies revealed maximum growth rates for some algae at DIN concentrations of about 0.5–0.8 µmol L^−1^ [98], [99]. However, the interactive effects of nutrient enrichment and herbivore exclusion on biomass (algal dry mass, organic C, N), and community composition on the light-exposed tiles showed the potential of nutrient enrichment on algal growth and composition.

Since microbial activity is enhanced by algal derived DOC [38], [39], we expected DOC concentrations in the water column to rise with increasing algal biomass. Surprisingly, no correlation patterns between DOC and biomass were detectable, possible due to a dilution effect. Nevertheless, a parallel study [100], conducted under the same conditions, resulted in treatment specific responses of coral associated bacterial communities.

#### Algae community structure

Filamentous algae benefited directly from herbivore exclusion since they are a main feeding substratum for many herbivores [101]–[104]. Concordant with a study by McClanahan et al. [47], filamentous algae on the light-exposed tiles grew best under the combined treatment with herbivore exclusion and elevated nutrient concentrations. The rapid response of the algae and the clearly distinguishable differences between the caged and non-caged treatments, together with a low abundance outside the frames (CJ pers. obs.) strongly suggest filamentous algae to be an indicator for herbivore overfishing in the investigated area [43], [44].

In contrast to a recent study by Jessen and Wild [55] in the Egyptian Red Sea, who found frondose brown algae within 4 wks after the start of a similar experiment, this algal group was not observed during the present study. Other studies from other oceans found frondose brown algae also within 4 months on their tiles [11]–[13], [50], [51], [86], though some of the examined substrates were likely affected by preconditioning. The absence of certain genera is likely due to a combination of seasonality and predation preferences [105], [106].

Concordant with Jessen and Wild [55] from the Red Sea, but contrary to other studies [11]–[13], [49], CCA cover was not found on the light-exposed tiles. Though Belliveau and Paul [11] and Smith et al. [13] preconditioned their tiles for 2 months, CCA appeared no later than 1 and 2 months respectively, indicating that settling and growth of CCA on the light-exposed tiles was inhibited in this study. The lack of CCA can be due to sediment trapping that can result in anoxic conditions coupled with decreased survivorship and recruitment of CCA [107]–[109]. The findings in the present study support this hypothesis: CCA grew on the shaded tiles where no filamentous algae dominated. The lower light conditions on the shaded tiles did not prevent CCA from growing, presumably due to their slow growing speed [110], [111].

Littler and Littler [45] proposed the Relative Dominance Model (RDM) that predicts benthic community structure in response to anthropogenic threats of overfishing (grazer reduction), elevated nutrients, and a combination thereof. Although, the present study was conducted in a limited time frame of 4 months, the results for this time period can neither confirm that CCA dominated in the high nutrient, high grazing treatment (shown by [12], [13] but not by [49]), nor the domination of frondose macroalgae under the combined treatments (shown by [13], [46], but not by [12], [47], [49]). However, following the model, filamentous algae predominated under low grazing levels (shown by [12], [13], [46]). Though, in contrast to the model, best conditions for filamentous algae in terms of biomass and cover were found in the combined treatment (shown by [47], but not by [13], [49]).

#### Differences between light-exposed and shaded tiles

This is the first study that compared the individual and combined effects of manipulated herbivory exclusion and nutrient enrichment on the reef algae community composition on light-exposed versus light-shaded tiles in coral reefs. The open space data (Figure 4A, Tables 3 and 4) showed that the tile surface colonization occurred faster on the light-exposed tiles than on the shaded tiles. Higher light availability, easier access for grazers, and the putative higher supply of recruits from the water column on the light-exposed tiles may be responsible for this difference.

CCA and non-coralline red crusts were found almost exclusively and were predominant on the shaded tiles, which have been fount to either enhance [14], [112]–[115] or impair coral recruitment [116]–[118]. The light-exposed tiles featured neither CCA nor invertebrate cover and only slight amounts of red crusts and red macroalgae. The lack of these algal groups on the light-exposed tiles could be due to out-competition by filamentous algae [119]. In contrast, (mucilaginous) cyanobacteria were the only group that grew exclusively on the light-exposed tiles and not on the shaded tiles (Figure 4G, Tables 3 and 4).

Our results corroborate the observation by Burkepile and Hay [12] that studies from deeper reefs (6–18 m, except [60]) showed minimal effects of nutrient enrichment on overall algal abundance and moderate effects on community structures. They supposed that these differences may resulted from high light conditions in shallow areas allowing macrophytes to take full advantage of nutrient enrichment and enable them to grow rapidly. However, if it is assumed that the lower light conditions on the shaded tiles simulate reduced water depths, the lower influence of nutrient enrichment there suggests an important role of water depth and light availability on the effect of nutrient enrichment [120], [121].

#### Seasonality

It remains unclear whether the algal community was still in the succession process or already at a final stage. In contrast to other successional studies that compared the effects of herbivore exclusion and nutrient enrichment (e.g. [12], [13]), filamentous algae on the light-exposed tiles declined after wk 4. Temperature is an important controlling factor for algae [122], [123] and the Central Red Sea is subject to strong seasonal temperature fluctuations [124]. However, ambient condition data from temperature loggers in this study (Figure S2) did not reveal correlating patterns of temperature and biomass, nor did CTD data of several parameters (turbidity, O_2_ saturation and Chl *a* along the transect) (data not shown). DIN concentrations in ambient and enriched treatments that peaked after wk 4 and declined afterwards may be an important factor.

#### Consequences & conclusions

Cascading negative effects have been reported when reef ecosystems were continuously exposed to overfishing of herbivores and increased nutrient concentrations. Algae can gain dominance over corals [22], resulting in less settling substrate for coral spat [26], [27], decreased herbivore grazing rates [25], and changes in C and N fluxes [36], [125]. Predicted climate change effects of ocean warming and acidification may further exacerbate these processes [126], [127].

The study underlines the importance of herbivory for the Red Sea, especially in the light of the relatively low herbivore biomass compared to other Indo-Pacific reefs and the high algal growth potential when herbivory was impeded. Surprisingly, macroalgal (here particularly filamentous algae) growth rates in the first 4 wks of this study greatly exceeded average patterns of the Indo-Pacific and even those of the Caribbean [75]. However, after 4 wks, coverage declined and resembled the average Caribbean cover (at 8 wk) and the lower Indo-Pacific values (at 16 wk). Our data suggest that the surveyed reef is not resistant against herbivore overfishing or a combination together with increased nutrient concentrations that has been simulated in this study. However, the potential compensatory feeding and the present herbivore biomass suggest that the benthic community is resistant against enhanced nutrient concentrations even when exceeding proposed thresholds.

## Supporting Information

Figure S1 **Study site.** Right panel shows position of the study area in the Red Sea. The circle on the left panel indicates the study site at the Northern tip of Al Fahal-reef, located about 13 km off the Saudi-Arabian coast.
(PDF)

Figure S2 **Temperature development at Al Fahal reef.** Daily average temperatures (± max/min) of the 16 experimental frames at 5 m water depths at Al Fahal reef over the study period from June to September 2011. Sampling times are indicated by vertical lines.
(PDF)

Figure S3 **δ15N isotopic signatures of homogenized cover of light-exposed tiles.** δ15N values (mean±SE) are shown for each treatment over 5 sampling times. Missing values of wk 1 and wk 4 resulted from insufficient algal material for analysis. P-values are calculated from 3-factorial ANOVA and originate from analysis across the whole study period (see Table S3 for full test results).
(PDF)

Table S1 **List of counted herbivorous fish.** Listed are families, species names, abundance (normalized to ind. m^−2^), and their biomass (normalized to g m^−2^).
(DOC)

Table S2 **List of counted sea urchins.** Listed are species names, abundance (ind. m^−2^), and their biomass (g m^−2^).
(DOC)

Table S3 **Results of the 3-factorial ANOVA of d15N isotopic signatures of cover from light exposed tiles.** Significant results are indicated by asterisks. Abbreviations: C = Cage, F = Fertilizer, T = Time.
(DOC)
