# Supplementary figures and images for: In-Situ Effects of Simulated Overfishing and Eutrophication on Benthic Coral Reef Algae Growth, Succession, and Composition in the Central Red Sea
Source: PLoS One. 2013 Jun 19;8(6):e66992. doi: 10.1371/journal.pone.0066992 (PMC3686771; doi:10.1371/journal.pone.0066992)

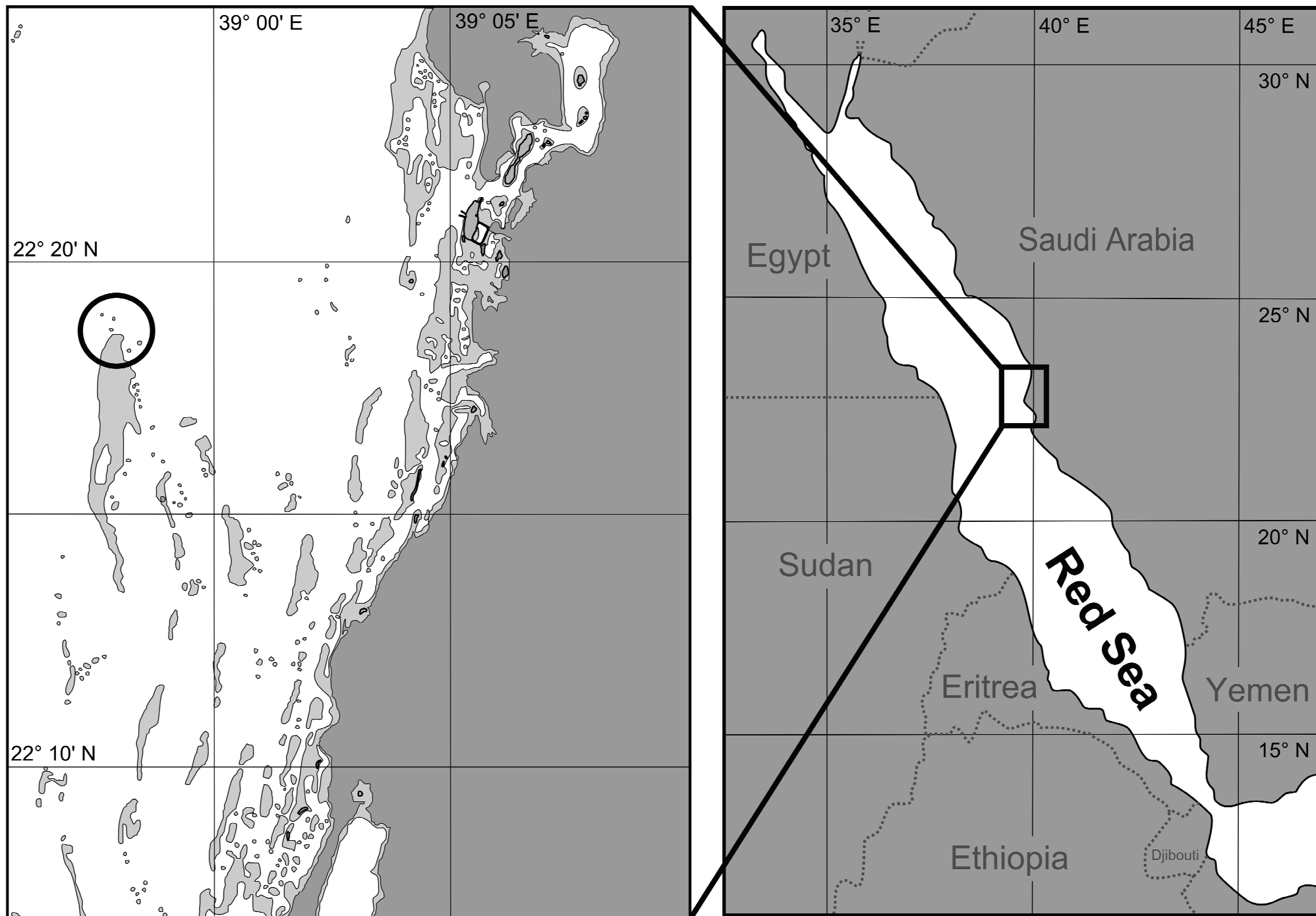

Supplement: Figure S1 — Study site. Right panel shows position of the study area in the Red Sea. The circle on the left panel indicates the study site at the Northern tip of Al Fahal-reef, located about 13 km off the Saudi-Arabian coast. (PDF) [file pone.0066992.s001.pdf]

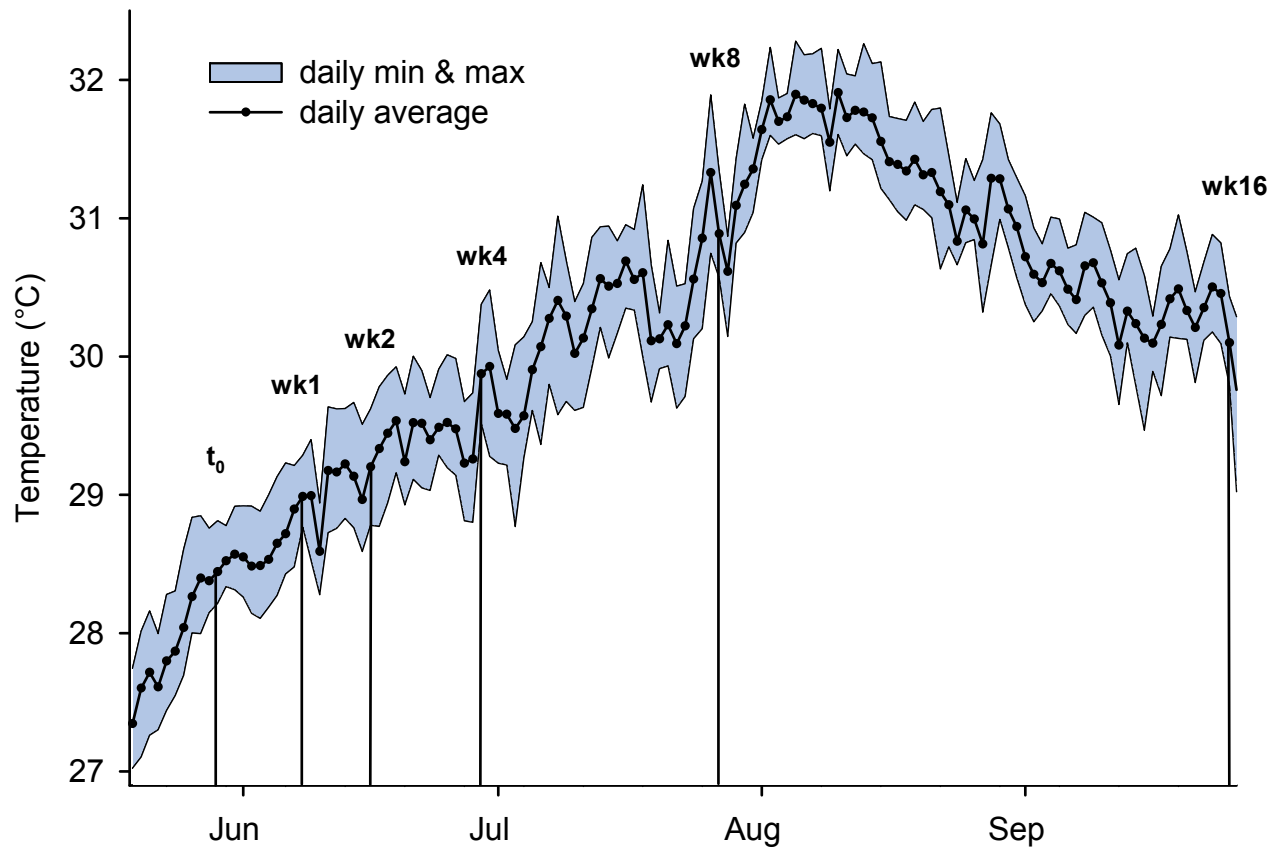

Supplement: Figure S2 — Temperature development at Al Fahal reef. Daily average temperatures (± max/min) of the 16 experimental frames at 5 m water depths at Al Fahal reef over the study period from June to September 2011. Sampling times are indicated by vertical lines. (PDF) [file pone.0066992.s002.pdf]

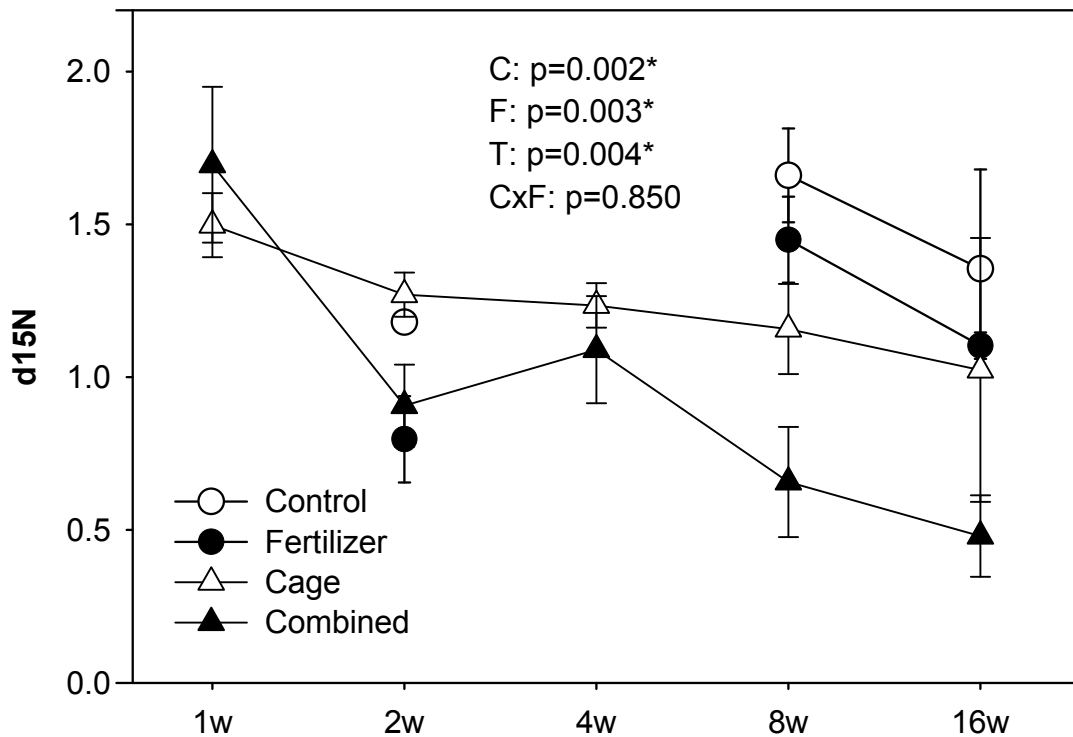

Supplement: Figure S3 — δ15N isotopic signatures of homogenized cover of light-exposed tiles. δ15N values (mean±SE) are shown for each treatment over 5 sampling times. Missing values of wk 1 and wk 4 resulted from insufficient algal material for analysis. P-values are calculated from 3-factorial ANOVA and originate from analysis across the whole study period (see Table S3 for full test results). (PDF) [file pone.0066992.s003.pdf]
