# Supplementary material for: In-Situ Effects of Simulated Overfishing and Eutrophication on Benthic Coral Reef Algae Growth, Succession, and Composition in the Central Red Sea
Source: PLoS One. 2013 Jun 19;8(6):e66992. doi: 10.1371/journal.pone.0066992 (PMC3686771; doi:10.1371/journal.pone.0066992)
Supplement: Table S1 — List of counted herbivorous fish. Listed are families, species names, abundance (normalized to ind. m−2), and their biomass (normalized to g m−2). (DOC) [file pone.0066992.s004.doc]

Table S1: List of counted herbivorous fish.

|  | Family | Species | Abundance | Biomass |  |
| --- | --- | --- | --- | --- | --- |
|  | Acanthuridae | all species | 2.70 | 9.77 |  |
|  |  | *Acanthurus nigrofuscus* | 0.049 | 0.57 |  |
|  |  | *Ctenochaetus striatus* | 0.086 | 1.54 |  |
|  |  | *Naso brevirostris* | 0.002 | 0.15 |  |
|  |  | *Naso elegans* | 0.021 | 5.12 |  |
|  |  | *Naso unicornis* | 0.005 | 0.86 |  |
|  |  | *Zebrasoma desjardinii* | 0.004 | 0.31 |  |
|  |  | *Zebrasoma xanthurum* | 0.026 | 1.22 |  |
|  |  |  |  |  |  |
|  | Ballistidae | *Balistapus undulates* | 0.009 | 0.69 |  |
|  |  |  |  |  |  |
|  | Chaetodontidae | *Chaetodon auriga* | 0.008 | 0.56 |  |
|  |  | *Chaetodon paucifasciatus* | 0.001 | 0.01 |  |
|  |  |  |  |  |  |
|  | Ostraciidae | *Ostracion cubicus* | 0.001 | 0.71 |  |
|  |  |  |  |  |  |
|  | Pomacanthidae | *Centropyge multispinis* | 0.011 | 0.14 |  |
|  |  |  |  |  |  |
|  | Pomacentridae | *Abudefduf sexfasciatus* | 0.076 | 0.54 |  |
|  |  | *Abudefduf vaigiensis* | 0.007 | 0.05 |  |
|  |  |  |  |  |  |
|  | Scaridae | all species | 0.071 | 8.89 |  |
|  |  |  |  |  |  |
|  | Siganidae | *Siganus stellatus* | 0.004 | 1.06 |  |
|  |  |  |  |  |  |
|  | Total |  | 0.380 | 22.42 |  |

Listed are families, species names, abundance (normalized to ind. m^-2^), and biomass (normalized to g m^-2^).
