# Supplementary material for: In-Situ Effects of Simulated Overfishing and Eutrophication on Benthic Coral Reef Algae Growth, Succession, and Composition in the Central Red Sea
Source: PLoS One. 2013 Jun 19;8(6):e66992. doi: 10.1371/journal.pone.0066992 (PMC3686771; doi:10.1371/journal.pone.0066992)
Supplement: Table S2 — List of counted sea urchins. Listed are species names, abundance (ind. m−2), and their biomass (g m−2). (DOC) [file pone.0066992.s005.doc]

Table S2: List of counted sea urchins.

|  | Species | Abundance | Biomass |  |
| --- | --- | --- | --- | --- |
|  | *Echinometra mathaei* | 0.64 | 9.98 |  |
|  | *Echinothrix calamaris* | 0.03 | 19.72 |  |
|  | *Eucidaris metularia* | 1.03 | 7.34 |  |
|  | *Heterocentrotus mammillatus* | 0.01 | 0.45 |  |
|  | Total | 1.71 | 37.49 |  |

Listed are species names, abundance (ind. m^-2^), and biomass (g m^-2^).
