# Supplementary material for: In-Situ Effects of Simulated Overfishing and Eutrophication on Benthic Coral Reef Algae Growth, Succession, and Composition in the Central Red Sea
Source: PLoS One. 2013 Jun 19;8(6):e66992. doi: 10.1371/journal.pone.0066992 (PMC3686771; doi:10.1371/journal.pone.0066992)
Supplement: Table S3 — Results of the 3-factorial ANOVA of d15N isotopic signatures of cover from light exposed tiles. Significant results are indicated by asterisks. Abbreviations: C = Cage, F = Fertilizer, T = Time. (DOC) [file pone.0066992.s006.doc]

Table S3: Results of the 3-factorial ANOVA of d15N isotopic signatures of cover from light exposed tiles.

|  |  | df | *F* | *P* |  |
| --- | --- | --- | --- | --- | --- |
|  | C | 1 | 11.05 | 0.002* |  |
|  | F | 1 | 9.80 | 0.003* |  |
|  | T | 4 | 4.53 | 0.004* |  |
|  | C x F | 1 | 0.04 | 0.850 |  |

Significant results are indicated by asterisks. Abbreviations: C=Cage, F=Fertilizer, T=Time.
